# Supplementary material for: Correlation between Pathogenic Determinants Associated with Clinically Isolated Non-Typhoidal Salmonella
Source: Pathogens. 2021 Jan 15;10(1):74. doi: 10.3390/pathogens10010074 (PMC7830680; doi:10.3390/pathogens10010074)
Supplement: Supplementary file 1 [file pathogens-10-00074-s001.pdf]

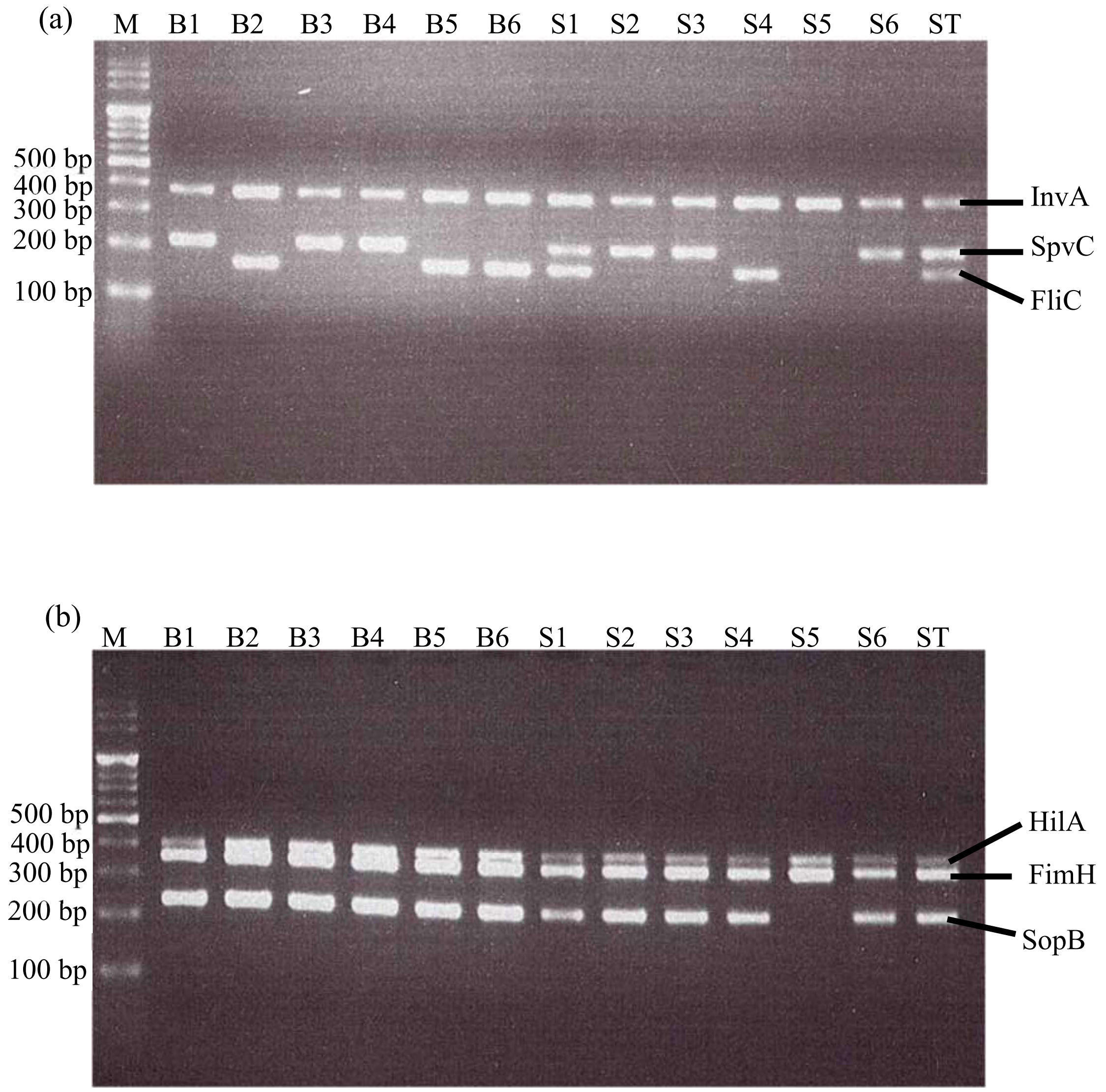

Figure 1. Agarose gels displaying (a) *FliC, SpvC, InvA* and (b) *SopB, FimH, HilA* amplicons produced by the multiplex PCR. Lane 1, M (100 bp ladder); Lane 2, B1; Lane 3, B2; Lane 4, B3; Lane 5, B4; Lane 6, B5; Lane 7, B6; Lane 8, S1; Lane 9, S2; Lane 10, S3; Lane 11, S4; Lane 12, S5; Lane 13, S6; Lane 14, *Salmonella* Typhimurium *LT2* (ST, positive control).

Table 1. Antimicrobial susceptibility summary from MIC test.

Drugs/ Iso-lates	AK	AMP	A/S	CZ	CPM	CMZ	CTX	CAZ	CTR	ETP	GEN	IPM	MRP	MI	PIT	TGC	COT
**B4**	R	S	S	R	S	R	S	S	S	S	R	S	S	R	S	S	S
**B26**	R	R	R	R	S	R	S	S	S	S	R	S	S	S	S	S	S
**B34**	R	R	I	R	S	R	S	S	S	S	R	S	S	R	S	S	S
**B74**	R	S	S	R	S	R	S	S	S	S	R	S	S	S	S	S	S
**B96**	R	S	S	R	S	R	S	S	S	S	R	S	S	S	S	S	S
**B104**	R	R	I	R	S	R	S	I	R	S	R	S	S	R	S	S	R
**B143**	R	S	S	R	S	R	S	S	S	S	R	S	S	S	S	S	S
**B163**	R	R	I	R	S	R	S	S	S	S	R	S	S	S	S	S	R
**B167**	R	R	I	R	S	R	S	S	S	S	R	S	S	R	S	S	R
**B168**	R	S	S	R	S	R	S	S	S	S	R	S	S	S	S	S	S
**B169**	R	R	R	R	S	R	S	S	S	S	R	S	S	S	S	S	R
**B182**	R	R	I	R	S	R	S	S	S	S	R	S	S	R	S	S	R
**B184**	R	S	S	R	S	R	S	S	S	S	R	S	S	R	S	I	S
**B187**	R	S	S	R	S	R	S	S	S	S	R	S	S	S	S	S	S
**B1673**	R	S	S	R	S	R	S	S	S	S	R	S	S	S	S	S	S
**B1697**	R	R	I	R	S	R	S	S	S	S	R	S	S	I	S	S	S
**B1701**	R	R	I	R	S	R	S	S	S	S	R	S	S	S	S	S	R
**B2127**	R	R	R	R	S	R	S	R	S	S	R	S	S	R	S	S	R
**B2146**	R	S	S	R	S	R	S	S	S	S	R	S	S	S	S	S	S
**B1**	R	S	S	R	S	R	S	S	S	S	R	S	S	S	S	S	S
**B2**	R	R	R	R	S	R	S	S	S	S	R	S	S	R	S	S	S
**B3**	R	S	S	R	S	R	S	S	S	S	R	S	S	S	S	S	S
**B4**	R	R	I	R	S	R	S	S	S	S	R	S	S	S	S	S	R
**B5**	R	S	S	R	S	R	S	S	S	S	R	S	S	S	S	S	S
**B6**	R	S	S	R	S	R	S	S	S	S	R	S	S	S	S	S	S
**S20**	R	S	S	R	S	R	S	S	S	S	R	S	S	S	S	S	S
**S30**	R	S	S	R	S	R	S	S	S	S	R	S	S	S	S	S	S
**S40**	R	S	S	R	S	R	S	S	S	S	R	S	S	S	S	S	S
**S50**	R	S	S	R	S	R	S	S	S	S	R	S	S	R	S	S	S
**S60**	R	S	S	R	S	R	S	S	S	S	R	S	S	S	S	S	S
**S70**	R	S	S	R	S	R	S	S	S	S	R	S	S	S	S	S	S
**S150**	R	R	R	R	S	R	S	S	S	S	R	S	S	S	S	S	R
**S160**	R	R	R	R	S	R	S	R	S	S	R	S	S	S	S	S	R
**S172**	R	R	I	R	S	R	S	S	S	S	R	S	S	R	S	S	R
**S179**	R	R	R	R	S	R	S	S	S	S	R	S	S	S	S	S	R
**S1627**	R	R	R	R	S	R	I	I	R	S	R	S	S	S	S	S	S
**S1630**	R	R	R	R	S	R	S	S	S	S	R	S	S	R	S	S	R

**S1635**	R	R	I	R	S	R	S	S	S	S	R	S	S	R	S	S	R
**S1645**	R	S	S	R	S	R	S	S	S	S	R	S	S	S	S	S	S
**S1647**	R	S	S	R	S	R	S	S	S	S	R	S	S	S	S	S	S
**S1690**	R	R	I	R	S	R	S	S	S	S	R	S	S	S	S	S	S
**S1691**	R	R	I	R	S	R	S	S	S	S	R	S	S	R	S	S	S
**S1707**	R	S	S	R	S	R	S	S	S	S	R	S	S	S	S	S	S
**S1715**	R	S	S	R	S	R	S	S	S	S	R	S	S	S	S	S	S
**S1**	R	R	I	R	S	R	S	S	S	S	R	S	S	S	S	S	S
**S2**	R	S	S	R	S	R	S	S	S	S	R	S	S	S	S	S	S
**S3**	R	R	I	R	S	R	S	S	S	S	R	S	S	S	S	S	S
**S4**	R	R	R	R	S	R	S	S	S	S	R	S	S	R	R	S	S
**S5**	R	R	I	R	S	R	S	S	S	S	R	S	S	I	S	S	S
**S6**	R	R	R	R	S	R	S	S	S	S	R	S	S	S	S	S	S

Isolates with B or S letter preceding ID name indicated the isolation sites Blood or Stool.

R: Resistant, I: Intermediate, S: Sensitive.

Amikacin (AK), Ampicillin (AMP), Ampicillin-Sulbactam (A/S), Cefazolin (CZ), Cefepime (CPM), Cefmetazole (CMZ), Cefotaxime (CTX), Ceftazidime (CAZ), Ceftriaxone (CTR), Ertapenem (ETP), Gentamicin (GEN), Imipenem (ETP), Meropenem (MRP), Minocycline (MI), Piperacillin-Tazobactam (PIT), Tigecycline (TGC), and Trimethoprim-Sulfamethoxazole (COT).

Table 2. *Salmonella* isolates virulence gene and mRNA expression levels.

Isolation site	Isolates ID	Serogroup	*FliC*	*SpvC*	*InvA*	*FimH*	*SopB*	*HilA*
Blood	4	C2	0.02±0.01	0.00±0.00	1.02±0.30	1.27±0.36	0.57±0.13	1.32±0.32
	26	B	0.00±0.00	0.00±0.00	0.86±0.13	1.34±0.41	0.43±0.08	1.44±0.37
	34	B	8.07±4.24	0.00±0.00	0.85±0.32	1.17±0.52	0.58±0.24	1.25±0.47
	74	D1	0.00±0.00	0.42±0.03	0.82±0.09	1.39±0.35	0.53±0.06	1.47±0.30
	96	C1	5.63±2.00	0.00±0.00	1.14±0.44	1.38±0.33	0.57±0.07	0.00±0.00
	104	C2	0.04±0.02	0.00±0.00	1.50±0.29	2.09±0.45	0.72±0.06	1.71±0.23
	143	B	0.00±0.00	0.00±0.00	1.24±0.39	1.43±0.31	0.77±0.12	1.55±0.24
	163	C2	0.04±0.02	0.00±0.00	0.85±0.13	1.20±0.44	0.50±0.08	1.43±0.34
	167	C2	0.03±0.02	0.00±0.00	1.08±0.18	1.23±0.17	0.69±0.09	1.62±0.28
	168	D1	0.00±0.00	0.44±0.08	1.14±0.21	1.29±0.26	0.56±0.08	1.34±0.26
	169	C2	0.03±0.01	0.01±0.01	0.83±0.12	1.32±0.37	0.46±0.04	1.10±0.30
	182	C2	0.01±0.01	0.00±0.00	1.12±0.22	1.87±0.58	0.65±0.14	1.60±0.36
	184	C2	1.55±0.99	0.00±0.00	0.97±0.04	1.49±0.35	0.66±0.04	1.50±0.13
	187	C1	0.01±0.00	0.00±0.00	1.01±0.11	1.43±0.10	0.60±0.02	1.51±0.09
	1673	C2	0.02±0.01	0.00±0.00	0.77±0.03	1.16±0.12	0.51±0.01	1.31±0.10
	1697	B	0.00±0.00	0.00±0.00	0.80±0.04	1.18±0.14	0.59±0.07	1.22±0.09
	1701	D1	0.00±0.00	0.44±0.06	0.92±0.17	1.11±0.23	0.52±0.11	1.23±0.33
	2127	E	0.35±0.34	0.00±0.00	1.00±0.09	0.94±0.18	0.55±0.05	1.12±0.14
	2146	B	0.00±0.00	0.00±0.00	0.82±0.13	1.28±0.13	0.58±0.04	1.43±0.02
	1517 (B1)	D1	0.00±0.00	0.38±0.07	0.71±0.08	1.08±0.15	0.50±0.10	1.15±0.19
	1596 (B2)	B	3.21±0.61	0.00±0.00	0.80±0.12	0.98±0.07	0.53±0.04	1.21±0.07
	1462 (B3)	D1	0.00±0.00	0.34±005	0.76±0.09	1.03±0.19	0.46±0.06	1.14±0.20
	1793 (B4)	D1	0.00±0.00	0.55±0.10	0.88±0.12	1.22±0.16	0.63±0.12	1.22±0.15
	1829 (B5)	B	0.72±0.02	0.00±0.00	1.08±0.11	1.53±0.18	0.65±0.03	2.86±1.52
	1833 (B6)	B	3.07±1.05	0.00±0.00	0.89±0.04	1.27±0.13	0.57±0.03	1.18±0.17
Stool	20	D1	0.33±0.33	0.90±0.32	4.96±4.03	5.62±4.19	0.80±0.09	1.94±0.38
	30	D1	0.00±0.00	0.20±0.20	1.28±0.29	1.74±0.35	0.68±0.06	1.91±0.42
	40	B	0.00±0.00	0.00±0.00	1.11±0.07	1.48±0.09	0.75±0.07	1.46±0.05
	50	C2	0.00±0.00	0.00±0.00	1.08±0.03	1.20±0.46	0.75±0.06	1.85±0.16
	60	D1	0.00±0.00	0.84±0.17	1.17±0.14	1.82±0.08	0.90±0.04	1.65±0.20
	70	D1	0.00±0.00	0.72±0.21	1.35±0.26	1.81±0.23	0.84±0.07	1.72±0.22
	150	E	0.00±0.00	0.00±0.00	1.08±0.15	1.80±0.32	0.74±0.09	1.48±0.16
	160	E	0.00±0.00	0.00±0.00	1.14±0.11	1.67±0.34	0.73±0.05	1.45±0.17
	172	C1	0.19±0.04	0.00±0.00	1.16±0.09	1.78±0.01	0.74±0.01	1.65±0.03
	179	E	0.00±0.00	0.00±0.00	1.01±0.05	1.88±0.12	0.65±0.03	1.55±0.14
	1627	D1	0.00±0.00	0.68±0.11	1.17±0.06	1.88±0.07	0.75±0.03	1.67±0.09
	1630	B	1.10±0.04	1.42±0.22	1.04±0.02	1.54±0.13	0.80±0.04	1.52±0.08
	1635	B	1.63±0.75	0.00±0.00	1.14±0.13	1.86±0.22	0.69±0.12	1.74±0.21
	1645	C1	0.00±0.00	0.00±0.00	1.07±0.11	1.75±0.14	0.66±0.11	1.51±0.13
	1647	E	1.01±0.13	0.00±0.00	1.00±0.03	1.65±0.08	0.76±0.00	1.44±0.09
	1690	D1	0.00±0.00	0.80±0.20	1.33±0.08	2.05±0.19	0.91±0.09	2.16±0.05
	1691	B	1.06±0.15	0.00±0.00	0.93±0.12	1.41±0.06	0.84±0.02	1.40±0.11
	1707	C2	0.00±0.00	0.00±0.00	1.20±0.04	1.75±0.14	0.81±0.11	1.60±0.02
	1715	C1	0.00±0.00	0.00±0.00	0.67±0.17	1.36±0.19	0.63±0.15	1.32±0.26
	1792 (S1)	B	0.72±0.16	1.24±0.25	1.08±0.16	1.79±0.25	0.92±0.09	1.51±0.09
	1785 (S2)	D1	0.00±0.00	0.91±0.40	1.36±0.26	1.61±0.55	0.53±0.27	1.43±0.25
	1794 (S3)	D1	0.00±0.00	0.83±0.26	1.44±0.09	1.74±0.30	1.16±0.18	1.91±0.15
	1795 (S4)	B	0.70±0.08	0.00±0.00	0.71±0.08	1.24±0.25	0.63±0.08	1.44±0.07
	1798 (S5)	B	0.00±0.00	0.00±0.00	1.13±0.07	1.74±0.05	0.88±0.08	1.62±0.03
	1762 (S6)	D1	0.00±0.00	0.60±0.09	3.58±2.78	1.28±0.29	0.57±0.14	1.78±0.55
Controls	*S. typhimurium*	NA	0.60±0.31	0.81±0.41	0.89±0.09	1.50±0.26	0.75±0.11	1.31±0.17
	E. coli	NA	0.00±0.00	0.00±0.00	0.00±0.00	0.00±0.00	0.00±0.00	0.00±0.00

Table 3. MIC breakpoint ranges.

	MIC (µg/ml)	
**Antimicrobial compounds**	**Minimal dose**	**Maximal dose**
Amikacin	≤8	-

Ampicillin	≤4	>16
Ampicillin-Sulbactam	≤4/2	>16/8
Cefazolin	≤2	>16
Cefepime	≤1	-
Cefmetazole	≤8	32
Cefotaxime	≤1	16
Ceftazidime	≤1	>16
Ceftriaxone	≤1	16
Ertapenem	≤0.25	-
Gentamicin	≤2	>8
Imipenem	≤0.25	-
Meropenem	≤0.25	-
Minocycline	2	>8
Piperacillin-Tazobactam	≤4	>64/4
Tigecycline	≤0.5	4
Trimethoprim-Sulfamethoxazole	≤0.5/9.5	>2/38
